# Computational modelling of cardiac resynchronization therapy in congenital heart disease: fantasy or the future?

**DOI:** 10.1093/europace/euae027

**Published:** 2024-01-24

**Authors:** Henry Chubb, Matteo Salvador, Alison L Marsden

**Affiliations:** Division of Pediatric Cardiology, Department of Pediatrics, Stanford University, 750 Welch Road, Palo Alto, CA 94304-5701, USA; Department of Bioengineering, Stanford University, Palo Alto, CA, USA; Institute for Computational and Mathematical Engineering, Stanford University, Palo Alto, CA, USA; Division of Pediatric Cardiology, Department of Pediatrics, Stanford University, 750 Welch Road, Palo Alto, CA 94304-5701, USA; Department of Bioengineering, Stanford University, Palo Alto, CA, USA; Institute for Computational and Mathematical Engineering, Stanford University, Palo Alto, CA, USA; Cardiovascular Institute, Stanford University, Palo Alto, CA, USA

**This editorial refers to ‘How to assess and treat right ventricular electromechanical dyssynchrony in post-repair tetralogy of Fallot: insights from imaging, invasive studies and computational modelling’, by M. Ložek *et al*., https://doi.org/10.1093/europace/euae024.**

In this issue, Ložek *et al*.^1^ explore the feasibility of tailoring a widely available biomedical modelling platform (CircAdapt) to create a ‘digital twin’ to quantify the projected impact of resynchronization of the right ventricle (RV). Prof Janoušek is a pioneer in the use of RV resynchronization in congenital heart disease (CHD), and the paper also serves as an succinct summary of many of the imaging concepts that the team has developed and adapted to this distinctive physiology.^2^ They outline some of the imaging hallmarks of RV electromechanical dyssynchrony, and in particular the unique implementation of the analysis of the systolic stretch index to the RV.^3^ However, it is the use of individualized modelling of cardiac resynchronization therapy (CRT) in CHD that is the most novel and is an important step forward in this nascent field.

For non-CHD patients, there is a long history of computational modelling of electrical dyssynchrony, with early studies published over 20 years ago.^4^ However, there are minimal data regarding the extension of these techniques to the more complex CHD anatomies. The results of the simulations presented by Ložek and colleagues are limited by the small sample size and limited number of physiological scenarios, at least in part due to the fact that fusion pacing in RV resynchronization limits the freedom to vary the V–V delay. Table 4 of their study illustrates that the majority of the predictions derived from the simulations are at least correct qualitatively, but there are inevitable quantitative discrepancies.

So, should the clinician feel that this is a glimpse of the future, or does it serve to temper expectation? When the role and place of CRT in the management of the CHD patient is discussed, there is often the suggestion that accurate computer models could provide some answers. However, the computational modelling techniques are almost limitless in potential complexity. This study serves not only as a stepping-stone towards this goal but also as a focus to review the strengths and limitations of computational modelling for this purpose.

In general terms, there are three main components to computational modelling: the inputs, the model itself, and the outputs. The authors included a relatively short list of inputs: chamber size, valve regurgitation, and a marker of mechanical activation delay (RV septal-to-lateral delay, derived from speckle tracking). However, there are many more inputs that are also measurable and modellable, and electrical activation and scar are likely to be particularly important. Higher resolution delineation of baseline electrical activation can be performed via electro-anatomic mapping (endocardial activation) or body surface potential mapping (epicardial activation), and myocardial scar is conventionally identified via late gadolinium enhancement on MRI. Also likely important, but more challenging, are fibre orientation and His Purkinje system distribution. Currently, these are generally only measurable *ex vivo* via diffusion tensor MRI or microCT. Yet more needs to be understood regarding factors such as whether inputs need to be individually tailored (vs. generic for each CHD anatomy) and when increasing resolution reaches negligible impact to the model output.

Computational models also vary greatly in computational workload and complexity. In this study, Ložek and colleagues used the CircAdapt model, which in its basic form is available for free download (https://www.circadapt.org) and can be run on a personal computer. It is a model representing chamber mechanics and closed-loop circulation and is a remarkable tool for demonstrating cardiac biology. CircAdapt allows a fixed set of parameters to be adjusted and models outputs that include pressure volume loops or valvular flows. The authors also likely used the Multipatch module, which additionally enables users to assign broad cardiac regions with activation time and mechanical properties to simulate regions such as scar, enabling a higher degree of ‘twinning’ with the subject.^5^ However, in order to achieve this degree of computational efficiency, CircAdapt is a zero dimensional, or lumped parameter, model which assumes a uniform distribution of the fundamental variables (volume, pressure, and flow) within any particular compartment (chamber, organ, or vessel) of the model at any moment in time. Some of the limits of the digital twin outcome accuracy are therefore almost certainly related to the simplicity of this macroscopic model.

Much more complex computer modelling techniques exist, and these higher dimensional models recognize the variation of important parameters in space. The modelling of multiple layers such as fluids, mechanics, and electrophysiology (‘multiphysics’) is highly complex, and it must be recognized that the computing burden far from trivial. In the days of concern that artificial intelligence is taking over the world, it is easy to forget that single physiological simulations can take many hours on super computers with hundreds of cores. The level of computational complexity is such that, in 2023, even GPU-accelerated three-dimensional multiphysics digital twins of the human heart still require upwards of 10 h for a single heartbeat (using eight Nvidia A100 devices, capable of ∼5 petaflops).^6^ Striving for computational efficiency will be crucial, likely with the aid of machine learning, as multiple iterations will also allow quantification of uncertainty related to accuracy of input parameters.^7^ We need to learn which input round-off errors amplify until they dominate the solution (a physiological ‘butterfly effect’), and which errors are tolerated.

And then what about the output? In this study, Ložek and colleagues chose to assess computed dP/dt, systolic stretch fraction, wasted work ratio, and exercise capacity (or cardiac output at fixed central venous pressure) as the output parameters for verification against clinical measurements. This is not unreasonable: hard outcomes such as survival or morbidity are many leaps of faith further down the line in terms of modelling. However, there remain concerns as to how clinically relevant these softer outputs are. Short-term measures (especially dP/dt) are relatively established as tools for optimization of CRT at implant, but the correlation with longer term outcomes such as reverse remodelling (let alone survival) is controversial.^8^ Furthermore, reverse remodelling itself is not only a clinically important outcome parameter but also a chronic feedback loop that is highly challenging to model.

So where does this leave us? Can computer modelling of CRT in CHD ever lead to clinically useful tools? Ložek and colleagues have demonstrated a path forward through this potentially limitless complexity. If relatively simple zero-dimensional tools can point in the right direction, then there is enormous promise. We currently have limited guidance as to which CHD patient will benefit from CRT, but it can be a powerful tool for survival for the correct patient.^9^ It is highly likely that we are withholding potentially life-saving therapy due to lack of data and the models initially need only be better than what little is currently available to guide the CHD cardiologist. From that point onwards, the sky is the limit. Supported by legislation such as the FDA Modernization Act 2.0, we need to strive for computational techniques that can be applied not only to drugs but also device therapies. With time, we will almost certainly find that three-dimensional, fully-coupled, electro-mechano-fluid mathematical models (or efficient surrogate models extracted from these ones) provide further and more reliable insights into CRT in CHD. However, like the statistics maxim of ‘as simple as possible but as complex as required’, if computer modelling can demonstrate clinically useful outputs, then the model may be complex enough. This study could prove to be the first small step in a great drive forward for CRT device therapy in CHD.

## Data Availability

There are no new data associated with this article.

## References

[1] Ložek M , Kovanda J, Kubuš P, Vrbík M, Lhotská L, Lumens J et al How to assess and treat right ventricular electromechanical dyssynchrony in post-repair tetralogy of Fallot: insights from imaging, invasive studies and computational modelling. Europace 2024:euae024. 10.1093/europace/euae024 38266248 PMC10838147

[2] Janousek J , Kovanda J, Lozek M, Tomek V, Vojtovič P, Gebauer R et al Pulmonary right ventricular resynchronization in congenital heart disease: acute improvement in right ventricular mechanics and contraction efficiency. Circ Cardiovasc Imaging 2017;10:e006424. 28877886 10.1161/CIRCIMAGING.117.006424

[3] Lumens J , Tayal B, Walmsley J, Delgado-Montero A, Huntjens PR, Schwartzman D et al Differentiating electromechanical from non-electrical substrates of mechanical discoordination to identify responders to cardiac resynchronization therapy. Circ Cardiovasc Imaging 2015;8:e003744. 26338877 10.1161/CIRCIMAGING.115.003744

[4] Usyk TP , McCulloch AD. Electromechanical model of cardiac resynchronization in the dilated failing heart with left bundle branch block. J Electrocardiol 2003;36:57–61. 14716593 10.1016/j.jelectrocard.2003.09.015

[5] Walmsley J , Arts T, Derval N, Bordachar P, Cochet H, Ploux S et al Fast simulation of mechanical heterogeneity in the electrically asynchronous heart using the MultiPatch module. PLoS Comput Biol 2015;11:e1004284. 26204520 10.1371/journal.pcbi.1004284 PMC4512705

[6] Viola F , Del Corso G, De Paulis R, Verzicco R. GPU accelerated digital twins of the human heart open new routes for cardiovascular research. Sci Rep 2023;13:8230. 37217483 10.1038/s41598-023-34098-8 PMC10203142

[7] Salvador M , Regazzoni F, Dede L, Quarteroni A. Fast and robust parameter estimation with uncertainty quantification for the cardiac function. Comput Methods Programs Biomed 2023;231:107402. 36773593 10.1016/j.cmpb.2023.107402

[8] Elliott MK , Mehta VS, Rinaldi CA. Pacing optimized by left ventricular dP/dt(max). Card Electrophysiol Clin 2022;14:223–32. 35715080 10.1016/j.ccep.2021.12.002

[9] Chubb H , Mah DY, Shah M, Lin KY, Peng DM, Hale BW et al Multicenter study of survival benefit of cardiac resynchronization therapy in pediatric and congenital heart disease. JACC Clin Electrophysiol 2023. 10.1016/j.jacep.2023.11.008 38206260

